# The inter-relationship of diversity principles for the enhanced participation of older people in their care: a qualitative study

**DOI:** 10.1186/s12939-020-1124-x

**Published:** 2020-01-28

**Authors:** Rajna Ogrin, Claudia Meyer, Arti Appannah, Sally McMillan, Colette Browning

**Affiliations:** 1 Bolton Clarke Research Institute, Level 1.01, 973 Nepean Hwy, Bentleigh, Victoria 3204 Australia; 2 Austin Health Clinical School, University of Melbourne, Heidelberg, Victoria Australia; 3 Biosignals and Affordable Healthcare, RMIT, Melbourne, Victoria Australia; 4 Department of Business Strategy and Innovation, Griffith University, Gold Coast, Australia; 5 LaTrobe University, Centre for Health Communication and Participation, Bundoora, Victoria 3086 Australia; 6 School of Primary and Allied Health Care, Monash University, Frankston, Victoria 3199 Australia; 7 LaTrobe University, Bundoora, Victoria 3086 Australia; 8 Bolton Clarke Clinical Learning Team, Level 1.01, 973 Nepean Hwy, Bentleigh, 3204 Australia; 9 School of Nursing and Healthcare Professions, Federation University, Ballarat, Victoria 3353 Australia; 10 International Institute for Primary Health Care Research, Shenzhen, China; 11 Research School of Population Health, Australian National University, Canberra, ACT, 0200 Australia

**Keywords:** Diversity, Aged care, Participation, Service delivery

## Abstract

**Background:**

The health and aged care workforce must understand and support the diverse needs of older people to enhance their care experience. We previously identified five principles of diversity training for this workforce: awareness of unconscious bias and prejudice; promotion of inclusion; access and equity; appropriate engagement; and intersectionality. This study aims to explore how these principles are considered from the perspectives of older Australians.

**Methods:**

Older people (≥65 years) receiving home care and nursing services based in Victoria, Australia were invited to participate in a home-based semi-structured interview about their experience of, or with, diversity. Interviews were thematically analysed using a priori categories based on our previous work on principles of diversity training, and themes were interpreted and expanded upon based on the participants’ experiences and understanding of diversity concepts and their care needs.

**Results:**

Fifteen older people (seven female, eight male), mean age 76 years (range 71–85 years), were interviewed. Five themes were drawn from the data. It was found that human connection through building (1) *trust and rapport* was highly valued as an approach by older people, crucial as a first step to understanding what is important to the older person. Identifying with (2) *intersectionality*, that is, the different intersecting aspects of who they are and their experiences was understood by the participants as an important framework to meet their needs. The participants were aware of (3) *unconscious bias and prejudice* by health professionals and its impact on their care. Participants also noted that (4) *promotion of inclusion through language* was important to for a positive relationship with the healthcare worker. The participants understood that to facilitate human connection, these four principles of human interaction were critical, underpinned by (5) *access and equity* of the system. A model articulating these relationships was developed.

**Conclusion:**

Health and aged care training should incorporate the five diversity principles to support older people to participate in their own care.

## Introduction

Worldwide, populations are ageing, and most people seek to age in place, that is, live in their homes for as long as possible [1, 2]. Ageing in place gives older people a sense of identity both through independence and autonomy and through caring relationships and roles in the places where they live [3]. Increasing age is associated with higher rates of complex, chronic conditions [4], leading to many older people needing support to age in place safely [5]. However, current systems are unable to support optimal wellbeing of older people and ageing in place. This is a result of a number of reasons: most health services were designed around acute care models, poorly aligning with the complexity and chronicity of issues associated with older age and the supports needed for older people to age in place [6]; there is endemic age-based discrimination [7, 8]; and limited understanding of the priorities and needs of older people [9].

To promote healthy ageing, the most effective approach involves systems that place older people in the centre of service delivery, where their needs and preferences drive their care and there is an integrated approach across service levels and types [1]. This approach aims to ensure that the changing and diverse needs of older people directs the care that is delivered. The World Health Organisation’s (WHO) Global Strategy and Action Plan on Ageing and Health includes driving for commitment to action on healthy ageing in every country, developing age-friendly environments, and aligning health systems to the needs of older populations [10]. To meet the health and social care needs of older populations, there needs to be an understanding of what these diverse needs are and how best to identify them. Further, the focus needs to move away from managing their specific chronic disease(s) towards what they need to support their everyday activities and aspirations for their daily lives [1].

By centring care on older people themselves, care providers’ focus should be directed at the individual with unique experiences, needs and preferences. Further, older people need to be considered within the context in which they live: that they are part of a family and a community. This entails respecting their dignity and autonomy, where a culture of shared decision-making is the norm [1]. The WHO plan assists key stakeholders in health and social care to understand, design, and implement person-centred and coordinated models of care, aiming to support older people to age with dignity and maintain wellbeing [11]. Countries around the world are striving to implement these ideals, including Australia [11, 12]. The WHO Integrated Care for Older People (ICOPE) package of tools was released on the International Day of the Older Person in 2019, specifically to support the implementation of this approach through this package [13]. Through this, recommendations were published on community-level interventions to manage declines in intrinsic capacity (ie. the composite of all the physical and mental capacities of an individual) of older people [14]. To achieve person-centred care in practice requires a community aged care workforce that can identify and respond to the health and care needs of all people with diverse characteristics, including older people [15].

In August 2013, Australia’s approach to aged care provision to support people to age in place underwent a significant change, moving from a fragmented system, to a system where consumers have choice, control and can access short term, episodic or ongoing services as needed [15]. Several other countries have implemented this approach, including the UK, the United States, Canada, Belgium and Netherlands [16]. While acknowledging variations, the underpinning principle of these reforms is self-directed care, offering the older person individual choice and control of their government-subsidised services, including where and how to spend their subsidy [17]. The framework for the changes is called Consumer Directed Care (CDC), which is defined as*. “... a way of delivering services that allows consumers to have greater control over their own lives by allowing them to make choices about the types of care and services they access and the delivery of those services, including who will deliver the services and when.”* [18]. Critically, a transition to this consumer-led approach requires the tailoring of care to meet an individual’s diverse needs [19]. Principles that ensure the dignity and human rights of each individual must underpin this approach, identifying the diverse characteristics and life experiences that may influence an individual’s care needs [19].

Currently, the care at home system supports around one million diverse older Australians each year [20]. It is anticipated that by 2050, over five million older Australians will access aged care services [15]. To ensure aged care supports are applicable and acceptable to all older Australians, in line with a human rights approach, the community aged care workforce will need to be responsive to the many diverse characteristics influencing the health and care needs of older people [15]. This will include responding to diverse characteristics as varied as age, gender, ethnicity, sexuality and disability. The authors of this paper have previously undertaken a meta-narrative review identifying five key principles of diversity training, essential in upskilling the health and aged care workforce in responding to the diverse needs of older people [21]. These principles are as follows:

1. **Awareness of unconscious bias and prejudice:** Encouraging individuals to self-identify their own unconscious, or implicit, bias [22]. Reduced engagement by older people with healthcare can occur because of unintentional judgments about older people by health and aged care staff [23].
2. **Promotion of inclusion:** Emphasising a focus on similarities between people rather than differences by health and aged care workers supports a sense of belonging [24]. Shared understanding and promoting respect is encouraged by an inclusive environment [25]. Language is critical, such as using “person with dementia,” rather than the derogatory “demented patient.”
3. **Access and equity:** The promotion of inclusive health care requires embedding access and equity in policy and practice. These components have wide-reaching effects for participation of older people in their health care [26]. Any deficits in these aspects should be identified by health and aged care workers who are trained to better understand the diverse needs of older people.
4. **Appropriate engagement:** Identifying meaningful characteristics of an individual supports the enhancement of participation in healthcare. Participation encompasses involvement in healthcare, sharing the power of decisions with health and aged care professionals [27]. Building trust and rapport, leading to an openness to discuss what is most meaningful, enhances engagement.
5. **Intersectionality:** This involves moving away from viewing people through a single lens, towards understanding the intersection of their various characteristics [28]. For example, the interplay of characteristics, such as an older woman, from a culturally and linguistically diverse background, living in a remote community with few services, will inform this woman’s ability to participate in healthcare in a meaningful way.

To ensure the needs of people requiring services are met and they are encouraged to participate in their healthcare requires them to share information about themselves. Human connection, that is, building trust and rapport, is critical to this sharing of information; a connection built on the perceptions and aspirations of the older person, of respect and recognition for their identity and equity in the health and social care partnership leading to their participation in healthcare [27]. This study aims to explore how these five diversity principles are considered by older people to improve participation of older people in their healthcare and promote human interaction between the older person and their aged care provider.

## Methods

This study was conducted and reported in accordance with the Consolidated Criteria for Reporting Qualitative Research [29]. This study is part of a larger multicomponent mixed methods project, ‘Promoting inclusive health care - Implementing a framework to support diversity in aged care’, funded by the Australian Government Department of Health. This project proposed to develop a national aged care approach to diversity awareness through the delivery and evaluation of a new Diversity Training Program delivered to the aged care workforce. The project focused on embedding the broader concept of diversity into the assessment of needs approach and other processes of the aged care system, empowering the aged care workforce to ensure that assessment is culturally appropriate, carer inclusive and promotes re-enablement.

The project originally aimed to develop, implement and evaluate the effect of diversity training on older community members receiving care from staff who had participated in the Diversity Education Program. Input from older community members was considered important by the research team; however, given the vast geographical expanse of the training across Australia, it was not logistically possible to access the experiences of community members who received care from the diversity trained staff. Instead, interviews were undertaken at the end of the project with older community members currently receiving care but who may not have received care from the diversity trained aged care workers, to ascertain the perception of diversity in the context of care delivery.

### Design

The design of this qualitative study is deductive [30], mapping participants’ responses to a priori questions around the five key diversity principles. A realist framework approach was taken, with the aim of providing a rich thematic description of the entire data set, to obtain a sense of important themes [30].

### Recruitment

**Fig. 1:**
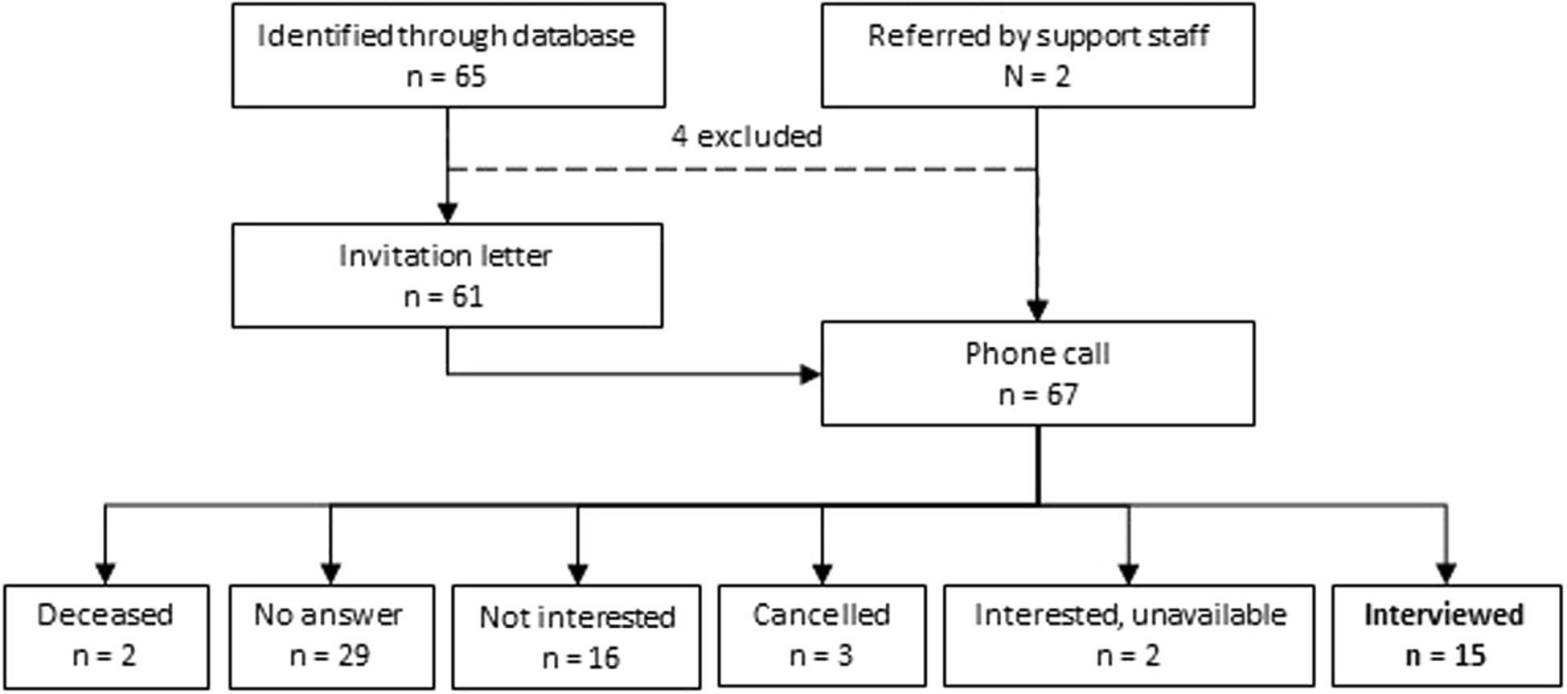
Recruitment process

Convenience sampling was used, with inclusion criteria of older people (≥65 years of age) receiving home care. Potential participants were identified from the Bolton Clarke database, the home care and nursing service agency based in Victoria, Australia from which they were receiving services and who had previously expressed interest in participating in research. An expression of interest letter was sent, with a follow up phone call from a researcher, made within a week of sending the letter. The recruitment process and numbers are shown in Fig. 1. If interested, a suitable appointment time was made by telephone and a Participant Information and Consent Form was mailed to the participant. Efforts were made to invite people from culturally and linguistically diverse backgrounds, using telephone interpreters for the initial invitation contact.

### Research team

The research team consisted of five female researchers, with research experience varying from limited, although having education and diversity education practical experience (SM), early career (AA, CM), mid-career (RO), to highly established (CJB). Three team members currently work within an aged and health care service organisation (RO, CM and SM), and two work in universities, although had previously worked in the same service organisation at the time the project was implemented (AA and CJB). The work backgrounds of the research team were also diverse, with academic psychology (AA, CJB) and clinical health in the fields of physiotherapy (CM), podiatry (RO), and nursing (SM).

### Data collection

Semi-structured interviews (see Table 1 for questions/prompts) were conducted in the participants’ own homes, at a mutually convenient time. The interviews were conducted by one member of the research team (CM), previously unknown to the participants. The researcher took care to understand her own biases and attitudes that may impact the interview. For example, having a belief that aged care workers, despite being time-pressured, should prioritise conversations with older people to understand them ‘as a person’. Interviews were audio recorded and transcribed verbatim by an external transcription service.

**Table 1:** Interview prompts used for the study participants

We’ve been running a project over the last couple of years about diversity and diversity in its broader sense. That is, not just cultural diversity, but any type of unique characteristic that someone might have and how that might impact their healthcare in some way. Perhaps you can begin by just telling me a bit about yourself and why you use [home nursing] services. Prompt topics if not covered in the discussion raised above – these were raised more in a conversation way, wended into discussion as the opening presented itself: • Is there anything that you would like to do but you feel like you can’t do at the moment? • Do you feel that the staff know what activities you would like to get back to? • Do you feel that you can talk to the doctors and nurses about what you want? • Do you feel that staff listen to you, and provide services in a way that suits you and your needs? • What positive characteristics do you think you’ve got to help you with your health? • Is there any characteristics about you that make it harder to get well? • Do you ever find it difficult to access health services? Physically getting there? • With services do you find that they are affordable for you? • Do you find you get enough information about your health care services and can read and understand it? • Have you ever felt that a service or a health professional has been unacceptable to you? • Have you experienced bias or prejudice during any health care encounters? • Do you feel that health professionals understand you as a person? • How would you like to be treated as a person in health encounters?	

### Data analysis

A theoretical thematic analysis was conducted within a realist framework, guided by the process described by Braun and Clarke [30]. This involved six phases: familiarization, generation of initial codes, searching, review and naming of themes, followed by report production [30]. However, firstly, two researchers (RO, CM) independently coded the transcripts, with quotes attributed to a priori categories of the five diversity principles [21] supported by NVivo software [31]. The following descriptions were used to guide the allocation of quotes to the diversity principles:

1. Awareness of unconscious bias and prejudice – quotes relating to unconscious or implicit bias on the part of the health and aged care worker, as identified along their care journey;
2. Access and equity – quotes relating to access to care;
3. Promotion of inclusion – quotes relating to interactions with health and aged care workers that supported a sense of belonging and inclusion in the care journey;
4. Appropriate engagement – quotes relating to supported discussions about what was meaningful for participation in care and how this engagement could be enhanced;
5. Intersectionality – quotes relating to their personal characteristics and identity, and the intersection of such, that may impact their participation in care.

Next, the researchers searched for repeated patterns of meaning, with prevalence related to the number of participants who raised the theme. The analysis sought to interpret the underlying ideas conveyed by participants, beyond simplistic interpretation of only words [30].

Three participants included their partners in the interview process. Partner input was minimal, predominantly serving to prompt the primary participant, and thus their data was subsumed with participant data for coding purposes.

### Reliability and validity

Credibility, transferability and confirmability were ensured so that the data and analysis would be trustworthy [32]. Credibility was demonstrated by establishing rapport and trust between the researcher and participants. Conflicting and contradictory comments were reported, with the provision of a rich description of the data ensuring transferability, allowing the researchers to draw inferences to their own experience. The research team was diverse, but included members embedded in the home nursing service. Reflexivity was therefore crucial to ensure that our beliefs and assumptions regarding participants views and the health system were not imposed on to the data, but rather allowed findings to inductively be drawn from the analysis. An audit trail of audio-recordings, verbatim transcriptions and the data analysis file ensured confirmability.

### Ethics

Ethics approval was granted by the Bolton Clarke Human Research Ethics Committee (project no. 164, approval no. 150005).

## Results

### Participants

Fifteen older people (seven female and eight male) were interviewed between April 10 and May 24, 2017. Average age was 76 years (range 71–85 years) and participants had received services from the agency for an average 7.7 years (range 0.2–15.7 years). Those who participated were no different based on age or gender to those who declined. The primary reason for in-home care was the management of leg wounds, infections and circulation problems (*n* = 11), with other issues including: medication management; neurological impairment and mental health conditions; spinal cord injury; and urinary catheterization. Three participants had migrant backgrounds and one was an ex-service member. Interviews were an average duration of 39 min (range 21–59 min).

### Thematic mapping to diversity principles

All data was coded to one of the five diversity principles. In addition, further interpretation of the meaning attributed by participants to these principles led to further themes beyond the five diversity principles: ***Intersectionality*** was interpreted by the researchers as a strong theme important to participants, reflecting the different aspects or characteristics of who they were, their identity, and how these characteristics intersected. ***Building rapport***, a human connection, was drawn out of the data by researchers as being critical in engaging with health and aged care workers. ***Unconscious bias*** and ***promotion of inclusion***, particularly through ***language,*** were found to impact the relationship between the health and aged care worker and older person, whereas the principle of ***access and equity*** related to system issues that supported or hindered participation in care. Each of these components are discussed in more detail below.

#### Intersectionality

All participants referred to their identity and their past experiences that have shaped who they are: complex, layered individuals. Their experiences were integral to their identity, and potentially relevant for participation in care. These included their past education, past employment, their family, their hobbies and activities important to them; a person, with a rich tapestry of experiences that made them who they were – not just an old person with a ‘wound’ or being a ‘diabetic’ or whichever health condition may have been the reason for referral.

*‘My university degree was in [majored in] applied mathematics, so that was really helpful, in helping with clear thinking and expression. All those things were set up in my early days’ (P04).*

*‘I was a professional cook, the cooking … My daughter has got six children and they used to come here - well, they still come but I used to cook. I used to love it. Bake and do all that sort of thing for them’* (*P08).*

*‘ … I am from Europe … I learned a lot. I was studying for 18 years only at schools’ (P14).*

This group of people have had many years of living, building up the unique mix of interests, physical and psychological needs and logistics, that is intersectionality, that blend for participation (or not) in healthcare. Their interviews provided depth and layers to these experiences, with participants eager to convey the different parts of their being, key to understanding who they are and what is important to their wellbeing.

*‘I took part in an experimental treatment of quadriplegics with music therapy, by a young woman who is a music therapist at the Austin. That was brilliant. The aim was to see what the effects of singing were on the respiratory capacity of a quadriplegic but also on the emotional state.*

*It was very uplifting. It was the best therapy of any kind that I’ve ever had. It was better than drugs. Of course, that came to an end. The young woman got her PhD but she got some special award too for the innovation of her treatment. So it was from that I joined the choir.*

*So I’d like to be able to join a choir. I’d like to take up singing and I’ve looked around for choirs but there’s nothing that I can find that suits. Usually they’re fairly late at night and in fairly inaccessible places’ (P10).*

#### Building trust and rapport

Fourteen of the 15 participants referred to what was important to them when engaging with health and aged care workers. The concept of developing a sense of connection with the health and aged care worker was raised by older people as very important for a positive care experience. This initial human connection is the beginning of trust and rapport, encompassing aspects of openness, authenticity, responsiveness, personalising the interaction to the individual, being empathetic and transparent. These aspects were identified as increasing providers’ capacity to interact and connect with older people in a meaningful and engaging way– that is, more than just the task-oriented care function. Each of these aspects are discussed in more detail below.

Openness was raised as a way of increasing the feelings of connection and sharing:

*‘You’ll more likely share with people, and feel more comfortable, if you get to know them as a person’ (P04).*

In contrast, some people wanted to maintain distance between the health and aged care worker and other aspects of their life, only sharing with them what was relevant to their health issue. The health and aged care worker was important in the context of needing to address a health concern, but not beyond this. Therefore, sharing of information required boundaries around openness:

*‘Yeah, they just treat you as a patient, they’re not [knowing] about your private life that much, I don’t think … Patient is a patient and that’s it - I mean, start to get involved in personal matters you might as well forget that, that’s silly’ (P02).*

Spending time with the person, and genuinely being interested in who they were was important for participants to feel that they could share more personal information. Being authentic was an important aspect of developing human connection and building rapport:

*‘Well I would think that just some informal questioning of a new client, new to them. Some of your staff may be pretty good at sitting down, making people feel comfortable. Have a cup of tea with them, and talk to them, just to start to understand them as a client’ (P04).*

*‘The people that get up my nose are the people that don’t show any interest’ (P07).*

Lack of responsiveness by some health and aged care workers to an individual, an apparent failure to listen, was distressing for some participants. Active listening, however, goes beyond merely listening, also encompassing an action in response to what an individual says:

*‘I was sort of angry because she didn’t listen to me. She was quite nice to talk to but she just wouldn’t listen. I said can you stop, please, I said it’s hurting a lot, she said I’m nearly finished. My god, it’s taken me three months to get my leg better’ (P06).*

The provision of care services are instigated by an older person (or someone on their behalf) to manage a health issue, but it moves beyond merely a task-orientated service. It means someone becomes a part of their life, spending time with them through regular care visits to their home. Human beings are social creatures, and any time that people come together there is some interaction that seeks more than just a transaction. Participants recognised that the health and aged care worker was there to address a health issue, but participants were adamant they were more than the health issue, they were an individual seeking a more personal interaction with acknowledgment of them as a person. Older people do not want to feel like they are ‘a task’, with no human connection beyond that task:

*‘ … she is very competent at her job. But I like to have something more with the carers than just their care function’ (P10).*

Participants were sensitive to the way health and aged care workers engaged with them, and sought to be treated with empathy, to be cared for. Being empathic is the health and aged care worker’s way of treating people in an understanding manner:

*‘You have two types of people … You’ve got the class of people that are extremely good and they care about what they’re doing, and you’ve got another class down here who don’t give a shit’ (P07).*

Participants were well aware they were seeking help to manage their health issue, but recognised they had autonomy and choice in their care. They were also aware that they needed a thorough understanding of what the care entailed and any further information that would assist them. Participants were willing to speak up, ask questions, challenge assumptions, and provide input into their care, from which a supported relationship with human connection can build:

*‘I’ve always gone by that. If you don’t understand anything, ask and see what it’s all about. It’s not going to hurt you. If they can’t answer you, well, they don’t know what they’re talking about’ (P02).*

*‘We have the right … to say no [to treatments]’ (P14).*

*‘I gave [smoking] away voluntarily myself, before the doctors got to tell me to give it away. Yeah, I woke up myself that it was doing me no good’ (P05).*

Human connection begins with the first, crucial interaction, where deceptively simple acts such as knowing the older persons’ name, and a few small details about them can be the foundation upon which rapport builds:

*‘I used to teach people to drive cars … I would make sure, before I pulled up or picked anyone up, that I knew what their first name was, what their surname was, their date of birth and a little bit about them, before I’d even met them … No matter what you do, if you’re trying to create a bond or a - yeah, a bond will do - they’re the things you need to start with. Following on from that, you need to know the person’s needs … and their wants’ (P07).*

Essential to having a viable and positive relationship with the health and aged care worker is the premise that the person is treated as someone who is a valuable member of the community in their own right. Participants were very clear that when interacting with health and aged care workers, they want to be treated with respect – like a human being, a normal person:

‘*Respect is something - people bandy it around as a word, but respect is something that’s earned, something is shown insomuch as how you approach people, how you interact with people in a respectful way and showing them the value of who they are and what sort of life they’ve lived in a lot of cases’ (P07).*

*‘How would I like to be treated? Just as a normal person. I’d like people to treat me as I treat them*’ (P07).

Participants understood they required assistance from the health and aged care worker, but felt it important that their strengths were acknowledged. A deficit may be present in one aspect of their lives, requiring assistance, but in many other aspects they continued to be independent, living their lives as they wish. An acknowledgement of strengths serves to build a foundation for a mutually respectful and caring partnership:

*‘Well, I’m very independent, I tell you. I prefer to walk myself. Even though I’m like a tortoise but I get there’ (P06).*

*‘I’m a bit of a smart alec. Don’t boss me around and tell me what to do... I’ve been my own person for too long [laughs]’ (P08).*

*‘I still think and work things out for myself. I don’t want to be just treated like somebody who is stupid. Do we give that impression when we’re old? Perhaps we do’ (P12).*

#### Awareness of unconscious bias and prejudice

Some participants felt that health and aged care workers, and the community more broadly, were biased, particularly related to the aspect of older age. They were concerned, and in some instances angry, that they were seen as less than who they are because of their age, with aspects overlooked due to their older appearance; when in fact they were individuals with a rich array of experiences, knowledge and skills. A clear message was expressed that older people wanted to be seen as a person, with their own personality, values, skills, and life experiences; and that this should not change with advancing age:

*‘Well I think sometimes when you’re older, that people think they’re doing you a favour. Well, I would think that that is irritating to people because you’re being treated as if you’ve lost your mind or - no, that’s not the right word. That you’re not aware of things. Well lots of old people, they are aware of things’ (P12).*

Participants with longstanding health issues guided new staff in the most effective and appropriate way to provide the necessary care. Participants, in addition to having autonomy and choice, valued being recognised for their knowledge and skills, their self-awareness and problem-solving abilities, that were important in navigating the care system and actively participating in their care:

*‘When new [staff] come, I say ‘if you need help I’ll help you’ … Because I see how my leg’s done every day and I know what they put on’ (P06).*

*‘I dare say [consumer directed care is] a case of arranging services that are available that provide that service within the cost. I think we’ll be able to work our way through it’ (P11).*

#### Promotion of inclusion through use of language

Facilitating inclusion of older people in their care involves interacting in a way that promotes a connection with their health and aged care worker. Communication is key, including the manner in which people talk to each other. Participants identified that language is important in shaping the relationship with the health and aged care worker:

*‘people can tell you what to do in a nice way and people will tell us what to do in a - trying to get the word - peremptory way. I can’t - as if what they believe themselves always to be right. That annoys me’ (P09).*

Language used by health and aged care workers has the ability to make older people feel that they are less than who they are, and promote feelings that it is purely because of their older age. Participants were living independently at home, with care services supporting them to do so. This independence was fiercely cherished, and interactions, through language, that a health and aged care worker has with an older person can impact their connection:

*‘Oh, well that they listen and take note of what you’re saying. I take great objection to be treated as if I don’t know what day it is …*. *I hate having my word - or my comments questioned, as if I don’t know what I’m talking about’ (P08).*

#### Access and equity

Access and equity, as identified by participants, related to system factors, including physical access to services, funding streams, time and organisational support. Physical access to appointments was a real challenge for many participants, with reliance on public transport, or taxis. Appointments were often a distance away, parking difficult or individual limitations impacting access:

*‘To an able bodied person it would be nothing, but to me it was a hell of a long way [to the clinic]. I’m not capable of doing that’ (P03).*

*‘I don’t think there’s enough services available to transport people who need it. We have these - what do they call them - taxis of multipurpose I think - who are solely there for transport people in wheelchairs and people who’ve got disabilities. You try and get one’ (P07).*

The most commonly raised system factor was time. Time was identified as a significant constraint within the healthcare system, creating a barrier in connecting and building rapport between the older person and the health and aged care worker. There was recognition that many community members needed care, with staff tasked with seeing an increasing number of older people, limiting the time they could spend with individuals. This reality had negative implications on care, as it limited the ability of health and aged care workers to get to know them and work better with them to support their health and wellbeing needs:

*‘They should have less work where they can … , spend more time getting to know the person that they’re dealing with. They haven’t got time for it’ (P07).*

*‘They’re always on the go these girls. To see what they do, how quickly they’ve got to do it and where they’ve got to go next - how they keep sane sometimes. They’ve just got too much work, in my opinion’ (P07).*

Participants understood the different levels of organisation required for the delivery of care. They also understood that this complex system requires navigation and interaction across the different providers and resources. Participants described the importance of communication and connection between themselves, their current health and aged care workers, managers and the wider healthcare system:

*‘The case manager, … they sent out an [Occupational Therapist] and under the [federal funding] arrangements, … organised an electric wheelchair. Yeah, … she organised that and I’ve used it ever since’ (P11).*

*‘I think it’s got to be in partnership. I think it’s got to be in partnership with the management, with the staff, and with the client. If you can develop that respect, and a bit of networking, and team work, I think is really important’ (P04).*

With the advent of consumer directed care, budgetary planning of service supports became necessary for participants, and they had to make hard decisions based on what was most important for their health and wellbeing. Resources are finite, particularly for those on a pension. These financial considerations impacted access and participation in care:

*‘Well yes because I liked to have a massage but a massage is $80. The - what was it - gym is $75. I had a personal trainer for half hour sessions a week. So that’s - what’s that? That’s $155 a week. But I have a pension and I have about $430 a week. So $150 out of that [laughs] becomes pretty impossible’ (P10).*

## Discussion

This paper confirms that the five diversity principles of awareness of unconscious bias and prejudice; promotion of inclusion; access and equity; appropriate engagement; and intersectionality underpin the needs of older people to participate in their care [21]. Older people have a need for openness, authenticity, empathy and respect as the precursor for participation in care to support them to age in place. The home care and nursing service agency at the centre of this study provides assistance to older people residing in the community; but, fundamentally, it is *how* this care is experienced that is crucial to older people. Interactions and relationships are very important: that is, human connection [33, 34]. Human connection is fundamental to achieving person-centred participation and thus, not surprisingly, is at the core of the care experience. The engagement with the care providers differed, depending on the level of and appropriateness of information they received in their interactions. Participants in this study acknowledged that good clinical care is essential, but it is the human connection with the care provider, alongside good clinical care, that appears to be critical for a positive care experience.

Below we first discuss how the five diversity principles for aged care worker education [21] are reflected in the views of our older participants. Next we describe how our findings add to the framework of person-centred participation in health care and the role of human connection developed by Thorarinsdottir and colleagues [27]. Based on this analysis we propose a new model of participation of older people in health and aged care.

### Diversity principles underpin care participation of older people

To promote a positive care experience, there was a need for health and aged care workers to be aware of their own ***unconscious biases and assumptions***, and to ***promote inclusion*** through language in their interaction with older people. In addition, the healthcare system more broadly must be underpinned with an ethos of ***access and equity***, to support older people to receive the care they needed. ***Intersectionality*** was woven through all participant interviews; who they were, based on their past education, employment, family, hobbies and other activities. There was a complex interplay of experiences that made participants who they were, and they wanted the health and care providers to acknowledge this complexity and treat them with respect. The experiences of older people in this study mirror those identified by studies included in a systematic review on experiences of home care by older people, where a number of themes were identified relating to the interactions between the care providers and the older person [35]. ***Appropriate engagement*** built on relationships and *rapport* developed between care givers and older people, which were of great significance to participants, with *trust* and mutual respect being very important. Older people needed to retain their sense of self and feel that they have autonomy. Our work adds to this literature by analysing the data against the previously developed diversity principles [21]. This will provide guidance to care providers for better interactions with older people, thereby supporting older people to participate in their care [35].

***Appropriate engagement*** through human connection has been identified via a framework analysis on person-centred participation in healthcare developed by Thorarinsdottir and colleagues [27]. This analysis identified human connection to be the foundation upon which individual participation in healthcare is built. Social engagement is an integral part of healthcare for older people [36] and, while a high level of knowledge and skill on the part of the professional is desired, these more technical aspects are mediated by worker attitudes and professional conduct [35]. Older people report positive interactions where healthcare providers were kind, respectful and caring; negative experiences were related to poor communication and lack of respect [35]. Good communication, necessary for positive human connection, leads to trust, mutual understanding, adherence to healthcare recommendations, social support and self-efficacy, all of which are associated with improved health [37]. The findings from this study were similar, but also identified the deeper aspect of embedded ***bias and prejudice*** that influences healthcare participation.

***Bias and prejudice*** are emotionally laden terms and are not usually associated with healthcare professionals. Health and aged care workers are well-positioned to promote inclusive healthcare, but need support to do so by changing the discourse of inclusive cultures and through challenging assumptions [38, 39]. It is difficult to understand the distinction between implicit (or unconscious) bias and prejudice, with this needing to be carefully built into training as, despite the best of intentions, all health and aged care workers carry unconscious bias, embedded through various life experiences [22, 40]. An awareness of this bias is critical to a positive healthcare experience, as made clear by the participants in this study. An undercurrent of bias may manifest as disrespect, ageism, lack of responsiveness and a de-valuing of their personal strengths and life experience. Older people want to be seen as valuable and active citizens and to be independent [9]. Older people want to feel respected, listened to (and heard) which can be achieved through respectful professional conduct, effective communication and advocacy on behalf of older people [35]. It is possible for health and aged care workers to identify their own unconscious bias and its impact on inclusive healthcare through education and training [40], critical for achieving human connection on which active participation in healthcare can be built.

Older people are not a homogenous group; instead, each older person is a unique mix of their physical and psychological make-up and life experiences. The intersection of these unique characteristics, or ***intersectionality***, contributes to the ability to connect with health and aged care workers, with each person having different needs and preferences. Older people’s experiences of care are affected by health providers’ levels of respect, impacting the older person’s sense of recognition and validation of self [35]. In the current study, older people acknowledged the need for help, but this did not equate to not being independent or being incapable of thinking for themselves. Support from services needs to tread the fine line to support older people to maintain, if not build, their independence [41]; enabling them by ‘doing with’, rather than ‘doing for’ [42, 43]. In fact, older people with complex needs would prefer not to accept a service if it would infringe on their ability to remain independent or to participate in social life [36]. It is critical to human connection and participation in healthcare that older people are viewed as autonomous individuals with a unique set of needs and preferences, who can be supported by the health and aged care workforce in an inclusive and respectful manner.

To promote human connection and participation in healthcare, there must be an ***accessible and equitable*** health and aged care system. The WHO promotes the Right to Health, including availability, accessibility and acceptability of health services, working with the social determinants of health as a basis [44]. This study highlighted the importance of physical accessibility, with lack of transport and limited mobility acknowledged as a barrier, well supported by the literature [36]. Equity encompasses acceptability of services, of which, according to participants, time is a crucial factor; time to build the human connection through development of rapport so that the needs and preferences of older people, according to the intersection of *their* diverse characteristics, can be identified. Organisational support and time as identified by participants are intrinsically linked and impact on the environment within which health and aged care providers can establish and maintain rapport, thus enhancing the acceptability of services. Older people recognise how these affect the quality of care that is delivered [45]. The Thórarinsdóttir & Kristjánsson (2014) framework, mentioned above, purports that organisational values must recognise and embed respect and equity, key to active participation in care [27]. The authors suggest that an inviting atmosphere or environment can help alleviate some of the potential psychological concerns of acceptability of services [27]. In various forms, accessibility and acceptability of services are crucial to an equitable health and aged care system.

### Proposing a new model for participation of older people in health and aged care

**Fig. 2:**
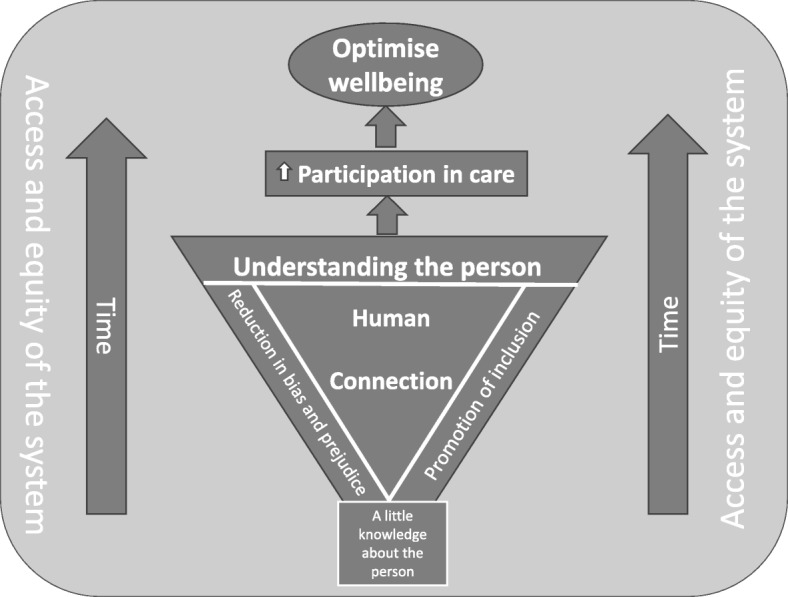
Model of participation of diverse older people in health and aged care

The five diversity principles highlighted within this paper do not exist in isolation, but rather are the sum total of the components to optimise participation in healthcare (see Fig. 2 for a diagrammatic representation). Based on the findings from the interviews and readings of the literature, we propose that human connection is key to understanding the individual needs and preferences of a person. With a little knowledge of the *person,* openness, responsiveness and the ever-elusive factor of time is critical to beginning this understanding, rather than only considering the *task* at hand. Feeling valued and respected for who they are, accounting for the intersection of their individual characteristics, increases the likelihood of participation in their own care, ultimately optimising their wellbeing. To support these interlinking diversity principles to enhance participation in care, health and aged care workers must receive education and training. Diversity training has most commonly existed as cultural competency training [21, 46] and, while this is important, it is not sufficient to understanding the intersection of diversity characteristics beyond cultural diversity. Training must focus on understanding human connection through building rapport, reducing bias and prejudice and promoting inclusion; and how these principles impact the older person.

### Strengths and limitations

This is the first study, building on previous work, to ask older community participants to consider diversity through the lens of the five identified principles. This study included only a small sample of people 65 years and older; our understanding would have benefited from a far wider spread of age, given that people from 65 years to beyond 100 years are a vastly heterogenous group. The principles that older people were asked to consider are new and conceptually challenging, making it difficult for older community members to articulate their thoughts. Further work is required to understand how the diversity principles impact the active participation in care of older community members beyond the building of human connection; including how best to draw this information from this population. Many attempts were made to include people from culturally and linguistically diverse backgrounds in this study, with this limitation needing to be addressed in a separate body of work. Other groups beyond cultural and linguistic diversity will need to be considered in further research (e.g. gender diversity, socio-economic differences and regional/rural versus metropolitan residency) to provide a greater understanding of the full breadth of perspectives of older Australians.

## Conclusion

The foundation of the active participation of older people in their healthcare is human connection. The five diversity principles lend themselves to broadening understanding of human connection: intersectionality to understand the complexity of individuals’ identities; building trust and rapport for positive interactions; communicating respectfully with an awareness of unconscious bias; and acknowledging the autonomy of the individual. Health and aged care workers will benefit from training in supporting human connection, designed to promote a positive care experience. Critically, the development of any such training should be informed by the experiences and perspectives of the older community members using these services.

## Data Availability

The datasets used and/or analysed during the current study are available from the corresponding author on reasonable request.
